# Cutaneous cryptococcosis in a patient with myelofibrosis receiving JAK‐inhibitor

**DOI:** 10.1002/jha2.339

**Published:** 2021-11-09

**Authors:** Jui‐Che Chen, Chang‐Tsu Yuan, Chien‐Chin Lin

**Affiliations:** ^1^ Hematological Oncology National Taiwan University Cancer Center Taipei Taiwan; ^2^ Department of Pathology National Taiwan University Hospital Taipei Taiwan; ^3^ Department of Internal Medicine National Taiwan University Hospital Taipei Taiwan

**FIGURE 1:**
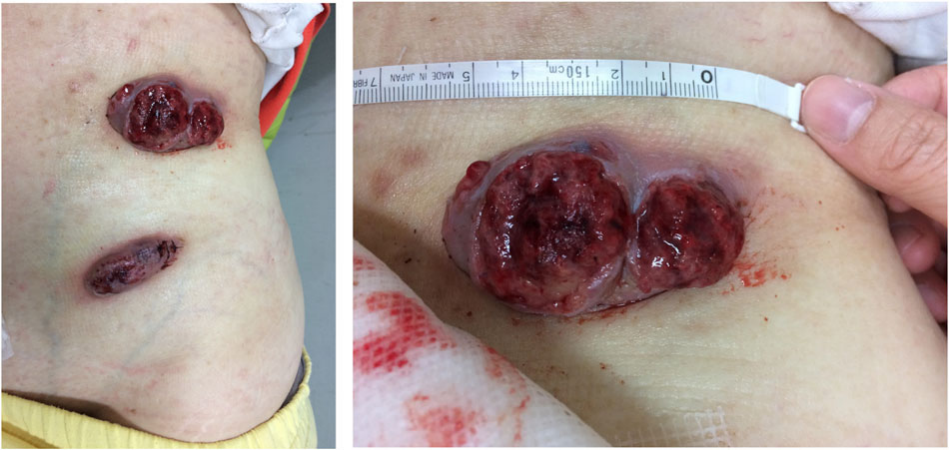
The two protruding masses on the abdomen

A 75‐year‐old woman diagnosed of post‐polycythemia vera myelofibrosis (post‐PV MF) with *JAK2*V617F mutation 11 years ago presented with two protruding, reddish, easily bleeding mass lesions over her left abdominal surface for 2 months (Figure 1). The lesions gradually enlarged to the size of 8 × 6 cm^2^ and 6 × 2 cm^2^, and progressed with pain and ulceration. Over the past 4 years, she received ruxolitinib treatment for MF‐related constitutional symptoms and splenomegaly with good control. Laboratory testing showed a white‐cell count of 7650 per cubic millimeter with 42% neutrophils and 5% blasts.

**FIGURE 2:**
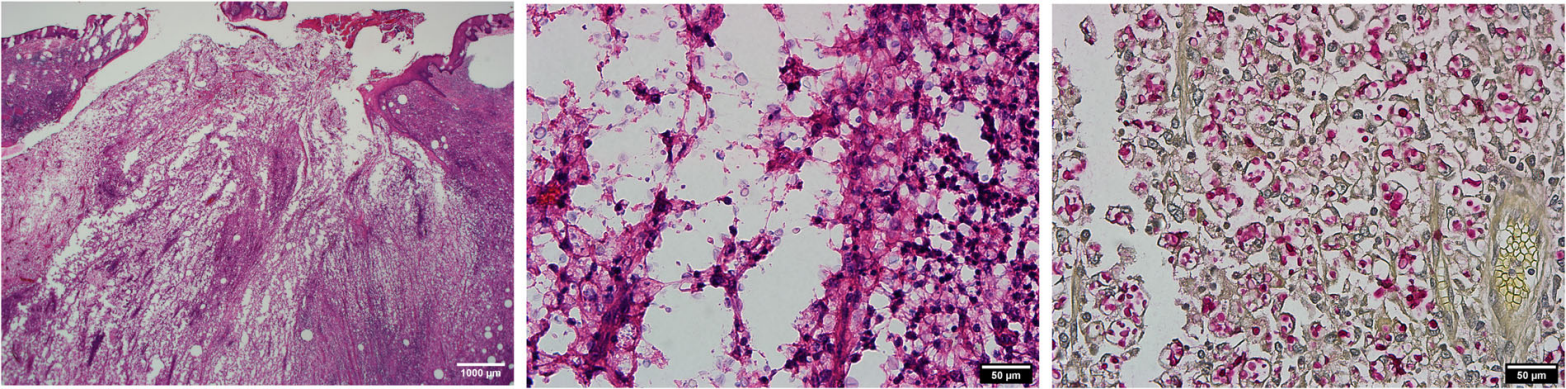
Excisional biopsy of the masses. **Left**: Hematoxylin and eosin stain, original magnification 20×. **Middle**: Hematoxylin and eosin stain, original magnification 400×. **Right**: Mucicarmine stain, original magnification 400×

Tumor excision biopsy revealed dense inflammation with cystic structure and a sinus tract‐like opening toward epidermis in low power view (Figure 2, left). In high power view, infiltrates of histiocytes, neutrophils, and yeast‐like microorganism with thick capsules in variable size were noted (Figure 2, middle). The capsules were highlighted by mucicarmine stain (Figure 2, right). Blood cryoptococcal antigen was positive at a titer of 1:256. Diagnosis of disseminated cryptococcosis with cutaneous involvement was confirmed.

Ruxolitinib, a janus kinases (JAK) pathway inhibitor, is currently the standard of treatment in higher risk myelofibrosis patients with significant benefits. However, correlation with slightly increased risk of atypical or opportunistic infection has also been reported. To our knowledge, this is the first case reporting cutaneous cryptococcosis in patient receiving JAK‐inhibitor.

